# Intra-session reliability of knee flexion-extension muscle strength monitored using a functional electromechanical dynamometer in female soccer players

**DOI:** 10.3389/fphys.2025.1542492

**Published:** 2025-02-25

**Authors:** Oscar Andrades-Ramírez, David Ulloa-Díaz, Bryan Alfaro Castillo, Vanessa Saavedra-Ibaca, Gustavo Muñoz-Bustos, Luis-Javier Chirosa-Ríos

**Affiliations:** ^1^ Department of Physical Education and Sport, Faculty of Sport Sciences, University of Granada, Granada, Spain; ^2^ Facultad de Educación y Ciencias Sociales, Universidad Andres Bello, Entrenador deportivo, Concepción, Chile; ^3^ Department of Sports Sciences and Physical Conditioning, Universidad Católica de la Santísima Concepción, Concepción, Chile; ^4^ Facultad de Humanidades y Educación, Universidad de Atacama, Copiapó, Chile; ^5^ Facultad de Salud y Ciencias Sociales, Departamento de Morfología y Función, Universidad de las Américas, Concepción, Chile; ^6^ Facultad de Salud, Escuela de Nutrición y Dietética, Universidad Santo Tómas, Iquique, Chile

**Keywords:** muscle strength, peak strength, dynamometer, reliability, female soccer

## Abstract

**Background:**

The aim of the study was to analyze the relative and absolute reliability of intra-session comparisons of three repetitions in a protocol for assessment peak muscle strength in a knee extension and flexion exercise in competitive female soccer players.

**Methods:**

The participants in this research are professional level female soccer players. Peak muscle strength was assessed with functional electromechanical dynamometry (FEMD) for the knee muscles with the following movements: knee flexion (FLE) and extension (EXT). Each movement was assessed at a speed of 0.4 m·s^-1^ unilaterally, recording peak muscle strength values in the concentric phase (CON) and an eccentric phase (ECC).

**Results:**

Null differences (ES < 0.19) were detected in the measurements of peak muscle strength of the extensors and flexors of the right and left knee in their concentric or eccentric phases. In the intra-set reliability measures, they reported acceptable absolute reliability (CV% < 9.71) and extremely high relative reliability (ICC = 0.92–0.98).

**Conclusion:**

In relation to the results of this study, it can be concluded that the FEMD presents a high relative and absolute intra-series reliability for the evaluation of muscle strength in knee extension and flexion in female soccer players. These reported antecedents may facilitate a more specific evaluation of the function of the muscles of the lower limbs.

## 1 Introduction

The planning and organization of reliable procedures for the evaluation and analysis of muscle strength has been of great interest in the fields of physical rehabilitation, sports medicine and sports performance (Janicijevic et al., 2023; Venegas-Carro et al., 2022; Donskov et al., 2021; Couto et al., 2023). More detailed analysis with greater accuracy in detecting changes resulting from training will provide coaches and professionals in strength and conditioning training with relevant information when organizing training and sports competition (McBride et al., 2022). Muscle strength is a physical quality that favors fast movements such as changes of direction, sliding, sprinting, jumping and contact with the opponent, actions characterized by high intensity that are fundamental during sports competition in both male and female soccer (Pecci et al., 2023; Barjaste and Mirzaei, 2018; Beato et al., 2021) requiring high levels of muscle strength in the lower limbs (Peñailillo et al., 2016), especially in the knee joint (Vargas et al., 2020; Gerodimos et al., 2023; Agresta et al., 2017). In addition, there is scientific evidence that muscle strength in the concentric phase and eccentric phase of the knee joint is a relevant measure for sports performance in soccer (Baroni et al., 2020).

In the last decade, women’s football has increased in popularity, development and professionalism; in many countries there are numerous professional clubs and leagues established in their respective countries (Randell et al., 2021; Federation Internationale the Football Association, 2020; Palka et al., 2023). When developing and monitoring appropriate training programs, a thorough understanding of the changes in players physical performance due to increased training volumes and competitive expectations of sports teams is necessary (Pardos-Mainer et al., 2021). Nowadays, sports competition is at a higher level and it is essential for technical bodies to develop accurate measurements of muscle strength with reliable and reproducible devices (Zemková and Hamar 2018; Rey et al., 2024).

There are currently devices on the market that can assess the muscle strength of any individual in a multi-joint manner with a wide range of movement of any section of the human body through a specialized cable without having the body fixedly attached to the device (Dvir and Müller, 2020). The Functional Electromechanical Dynamometry (FEMD) is a new device that allows for the free assessment of an individual’s muscle strength (del-Cuerpo et al., 2023). A unique feature is that it can operate in two modes: a) static without displacement (isometric mode and vibratory isometric mode), b) dynamic with displacement (conical, inertial, elastic, kinetic and tonic) measuring by a stable and variable resistance/velocity (Andrades-Ramírez et al., 2024a).

Previous research has examined the reliability of an FEMD between sessions. In the study by Martinez-Garcia et al. (2020), it was observed that the absolute reliability was “acceptable” and the relative reliability was “extremely high” of an FEMD, for mean muscle strength in the assessment of the strength of the internal rotator muscles and the external rotator muscles of the shoulder in a standing position at a speed of 0.60 m·s^-1^ and 0.30 m·s^-1^ in thirty-two university students in the concentric and eccentric phase. In the study by García-Buendía et al. (2024), they observed an absolute reliability between CV = 7.56–18.76% and relative between ICC = 0.76–0.94, in a battery of evaluations of muscular strength in flexion, extension, horizontal abduction and horizontal adduction of the shoulder using an FEMD.

In the study by Sánchez-Sánchez et al. (2021), they studied the reliability of muscle strength in an eccentric phase hamstring swing exercise with an FEMD in nineteen male soccer players, observing a “high” absolute reliability and an “extremely high” relative reliability in mean muscle strength for a displacement at a speed of 0.4 m·s^-1^. And for peak muscle strength at speeds of 0.2 m·s^-1^ a high absolute reliability and an “extremely high” relative reliability were obtained (ICC = 0.91). However, to date, relative and absolute reliability studies with FEMD have only reported inter-session comparison assessments of muscle strength, and no study has been conducted analyzing relative and absolute reliability for an intra-session for a peak strength exercise with an FEMD and only one study (Andrades-Ramírez et al., 2024b) has been conducted in a female athlete population with the device.

According to the background presented, the objective of the study was to analyze the relative and absolute reliability of intra-session comparisons of tree repetitions in a protocol for assessment peak muscle strength in a knee extension and flexion exercise in professional female soccer players. The hypothesis of this study is that this method of assessing muscle strength will be a reliable method in an intra-session measurement with three repetitions of strength in a concentric and eccentric displacement phase for the knee flexion and extension musculature with an FEMD device. This will provide better information when assessing the performance of female soccer players.

## 2 Materials and methods

### 2.1 Study design

A three-fold repeated measures design was used to analyse the reliability of a peak muscle strength test in FLE-EXT knee joint movements with FEMD. Participants in this study were presented with two familiarization sessions. Session 1: was used to assess anthropometry and familiarize participants with the use of the FEMD device, session 2: experimental testing was performed with FEMD. A non-probabilistic sampling was carried out due to cohabitation. Peak muscle strength assessments were performed at the Controlled Natural Movement Evaluation Laboratory of the Universidad Católica de la Santísima Concepción Chile under ambient conditions of temperature 21°C and humidity 60% humidity.

### 2.2 Participants

The participants in this study were twenty-one professional female soccer players (age = 18.65 ± 0.74 years; Weight = 57.53 ± 6.74 kg; Height = 1.60 ± 0.04 m; BMI = 22.21 ± 2.14 kg/m2; Training volume: 6 days/week and 2 h/day), with no experience in the use of FEMD. Participants signed their informed consent and voluntarily participated in the study. The participants (a) must have more than 5 years of experience in the sport of soccer, (b) Not have any musculoskeletal injury that prevents the correct evaluation of muscle strength in your lower limbs, (c) Participants who did not complete the evaluation protocol were excluded from the analysis (n = 1). Before starting the evaluation, participants in this study were verbally informed about the purpose and potential risks of the experimental procedure. The approval of the study protocol was carried out by the ethics and research committee of the Universidad Católica de la Santísima Concepción, Chile No. 01/2024 (approved 01 April 2024) following the recommendations of the Declaration of Helsinki (World Medical Association, 2013).

### 2.3 Materials

Participants in this study assessed their peak muscle strength with a FEMD (Dynasystem, Model Research, Granada, Spain). The device has an accuracy of 3 mm for displacement, 100 g for the detected load, a sampling frequency of 1,000 Hz and a velocity range between 0.05 m·s^−1^ and 2.80 m·s^−1^. It controls and regulates the precision of both force and linear velocity by means of a 2000 W electric motor. A wide variety of movements in different anatomical planes can be evaluated. The device can deliver a wide variety of resistance/velocity stimuli for the evaluation of muscular strength in its different manifestations. Displacement and velocity data are collected with a 2,500 ppr encoder that is attached to a roller inside the device. Samples from the different sensors are obtained at a frequency of 1 kHz. A load cell detects the tension applied to the rope, and the resulting signal is passed to an analogue-to-digital converter with 12-bit resolution.

### 2.4 Assessment of maximal muscle strength

The session begins with a general warm-up consisting of: (a) 5 min of cycling and static rowing at a heart rate reserve of 60%, plus 10 min of joint mobility exercises and dynamic stretching of the lower limbs, ending with muscle strength exercises for the hamstrings (bridges) and quadriceps (barbell squats). (b) The specific 10-minute warm-up, with assessment of submaximal muscle strength at the selected speed with a load of 5% of body weight as a load for the flexor muscles and 10% for the extensor muscles of the knee joint in a sitting and prone position (posterior muscle strength assessment position), 2 sets of 3 repetitions and 3 min of rest between sets with an FEMD were implemented. Study participants were asked to perform their maximum effort for each of the assessments. Movements were assessed unilaterally at a velocity of 0.4 m·s^-1^. Peak muscle strength was recorded with FEMD after set.

**FIGURE 1:**
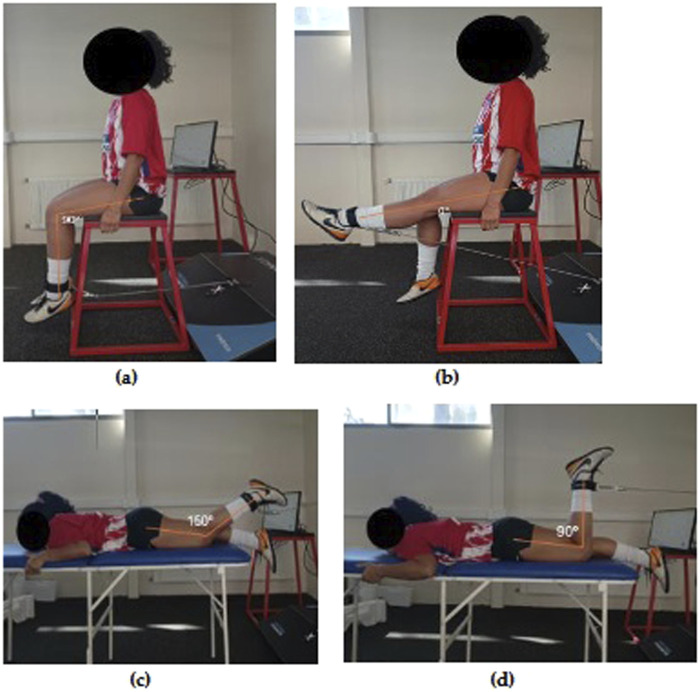
Assessment of peak muscle strength in knee extensor muscles: **(A)** initial position; **(B)** final position. Assessment of peak muscle strength in knee flexor muscles **(C)** initial position; **(D)** final position.

A functional range was used for each participant of extension from 90° to 0° and flexion from 150° to 90° which were measured with an analogy goniometer. Assessments were performed in the seated position and prone position (Figure 1). For each of the movements evaluated, 3 repetitions were used. The evaluations had a 5 min break between series and the order of evaluation of muscle strength was: (1) right knee extension (RKE), (2) left knee extension (LKE), (3) right knee flexion (RKF) and (4) left knee flexion (LKF).

### 2.5 Statistical analysis

Descriptive statistical models were used to calculate means and standard deviations (SD). The normal distribution of the data was analysed using the Shapiro-Wilk statistical model. Standardised mean differences (effect size for repeated samples) were used to compare the magnitude of loading between test replicates. The criteria for interpreting the magnitude of the effect size (ES) were as follows: very large (>2.00), large (1.20–2.00), moderate (0.60–1.19), small (0.2–0.59) and null (<0.20) (Hopkins, 2015). The absolute reliability of peak muscle strength was measured using the standard error of measurement (SEM), in addition to the use of the coefficient of variation (CV). For the evaluation of relative reliability, the intraclass correlation coefficient (ICC) model was used. The criteria used to classify reliability were acceptable (CV ≤ 10%) and high (CV ≤ 5%) (Weir, 2005). For the classification of relative reliability (ICC), values close to 0.9 were used extremely high, 0.7 very high, 0.5 high, 0.3 moderate and 0.1 low (Brady et al., 2020). The agreement and the calculation of systematic bias of the FEMD peak muscle strength measures were analysed by constructing Bland-Altman plots, and the 95% limits of agreement between the muscle strength measures were established (Boehringer and Whyte, 2019). The Bland-Altman graph presents the Heteroscedasticity of the errors with limits of agreement of 95% [LoA] = bias ±1.96 standard deviation. It was specified by the adjustment values for the coefficient of determination (*R* ^2^) < 0.1 (Atkinson and Nevill, 1998). Calculate the correlation of the outcome variables between the three repetitions of a muscle strength assessment session, the Pearson correlation coefficient (r) statistical model was used. The criteria for classifying the magnitude of r were perfect (1.00), almost perfect (0.90–0.99), very large (0.70–0.89), large (0.50–0.69), moderate (0.30–0.49), small (0.10–0.29), and null (0.00–0.09) (Hopkins et al., 2009). For analysis of the calculations of the statistical models, the use of a 95% confidence interval was defined. The accepted statistical significance was set at p < 0.05. All reliability assessments used a custom spreadsheet (Hopkins, 2015), the other statistical analyses presented in this study were created using the JASP software (version 0.16.4).

## 3 Results

Null differences (ES < 0.19) were detected in the measurements of peak muscle strength of the extensors and flexors of the right and left knee in their concentric or eccentric phases. In the intra-session reliability measures, an acceptable absolute reliability (CV% < 9.71) and an extremely high relative reliability (ICC = 0.92–0.98) were obtained for all maximum muscle strength evaluations as shown in the following Table 1.

**TABLE 1:** Assessment of peak muscular strength in extension and flexion of the right knee in the concentric and eccentric phase.

	Mean ± SD (N)		ES	SEM	CV%	ICC
Right knee extension						
	Rep 1	Rep 2		(95% CI)	(95% CI)	(95% CI)
Concentric	163.38 ± 44.33	169.00 ± 45.54	0.12	1.46 (1.07–2.30)	0.88 (0.64–1.39)	0.97 (0.94–0.99)
Eccentric	288.06 ± 92.94	292.46 ± 103.67	0.04	19.82 (14.37–31.93)	6.83 (4.95–11.00)	0.96 (0.90–0.98)
	Rep 1	Rep 3				
Concentric	163.38 ± 44.33	164.98 ± 44.59	0.04	2.59 (1.88–4.17)	1.58 (1.14–2.54)	0.98 (0.96–0.99)
Eccentric	288.06 ± 96.18	288.34 ± 97.18	0.01	27.20 (19.72–43.82)	9.44 (6.84–15.20)	0.97 (0.79–0.97)
	Rep 2	Rep 3				
Concentric	169.00 ± 45.54	164.98 ± 44.59	0.09	2.70 (1.96–4.35)	1.62 (1.17–2.60)	0.98 (0.94–0.99)
Eccentric	292.46 ± 103.67	288.34 ± 97.18	0.04	24.70 (14.90–39.79)	8.50 (6.16–13.69)	0.94 (0.85–0.98)
*Left knee extension*						
	Rep 1	Rep 2				
Concentric	161.06 ± 40.61	167.60 ± 43.08	0.16	6.58 (4.77–10.60)	4.00 (2.90–6.45)	0.98 (0.94–0.99)
Eccentric	283.06 ± 93.47	280.32 ± 100.98	0.04	22.42 (16.26–36.13)	7.95 (5.76–12.81)	0.95 (0.86–0.98)
	Rep 1	Rep 3				
Concentric	161.06 ± 40.61	166.03 ± 42.19	0.12	5.36 (3.89–8.64)	3.28 (2.38–5.28)	0.98 (0.96–0.99)
Eccentric	283.06 ± 93.47	268.46 ± 100.45	0.16	26.15 (18.96–42.13)	9.47 (6.86–15.25)	0.93 (0.81–0.97)
	Rep 2	Rep 3				
Concentric	167.60 ± 43.08	166.03 ± 42.19	0.04	3.51 (2.54–5.65)	2.10 (1.53–3.39)	0.98 (0.96–0.99)
Eccentric	280.32 ± 100.98	268.46 ± 100.45	0.12	26.63 (19.31–42.91)	9.71 (7.04–15.64)	0.94 (0.82–0.97)
*Right knee flexion*						
	Rep 1	Rep 2				
Concentric	72.18 ± 20.05	76.09 ± 20.38	0.19	4.31 (3.12–6.64)	5.81 (4.21–9.37)	0.96 (0.88–0.98)
Eccentric	145.06 ± 50.58	147.48 ± 53.40	0.05	6.83 (4.95–11.01)	4.67 (3.39–7.52)	0.98 (0.95–0.99)
	Rep 1	Rep 3				
Concentric	72.18 ± 20.05	76.61 ± 21.34	0.12	4.36 (3.16–7.02)	5.86 (4.24–9.43)	0.96 (0.89–0.98)
Eccentric	145.06 ± 50.58	140.78 ± 44.16	0.09	12.40 (8.99–19.98)	8.68 (6.29–13.98)	0.94 (0.83–0.97)
	Rep 2	Rep 3				
Concentric	76.09 ± 20.38	76.61 ± 21.34	0.02	4.38 (3.18–7.06)	5.74 (4.16–9.25)	0.96 (0.89–0.98)
Eccentric	147.48 ± 53.40	140.78 ± 44.16	0.14	12.64 (9.17–20.37)	8.77 (6.36–14.13)	0.94 (0.83–0.97)
*Left knee flexion*						
	Rep 1	Rep 2				
Concentric	72.34 ± 19.71	77.03 ± 20.97	0.18	3.19 (2.32–5.14)	4.28 (3.10–6.89)	0.98 (0.94–0.99)
Eccentric	139.61 ± 44.41	145.19 ± 48.55	0.12	7.73 (5.61–12.46)	5.43 (3.94–8.75)	0.97 (0.93–0.99)
	Rep 1	Rep 3				
Concentric	72.34 ± 19.71	76.29 ± 20.72	0.19	3.28 (2.38–5.29)	4.42 (3.20–7.12)	0.98 (0.93–0.99)
Eccentric	139.61 ± 44.41	138.66 ± 39.93	0.02	12.18 (8.83–19.61)	8.75 (6.34–14.10)	0.92 (0.79–0.97)
	Rep 2	Rep 3				
Concentric	77.03 ± 20.97	76.29 ± 20.72	0.04	3.81 (2.76–6.14)	4.97 (3.60–8.01)	0.97 (0.91–0.99)
Eccentric	145.19 ± 48.55	138.66 ± 39.93	0.15	12.54 (9.09–20.20)	8.83 (6.40–14.23)	0.93 (0.83–0.97)

Rep, repetition; N, newton; SD, standard deviation; ES, Cohen’s d effect size; SEM, standard error of measurement; CV%, coefficient of variation; ICC, intraclass correlation coefficient; 95% CI, 95% confidence interval; s, seconds.

**FIGURE 2:**
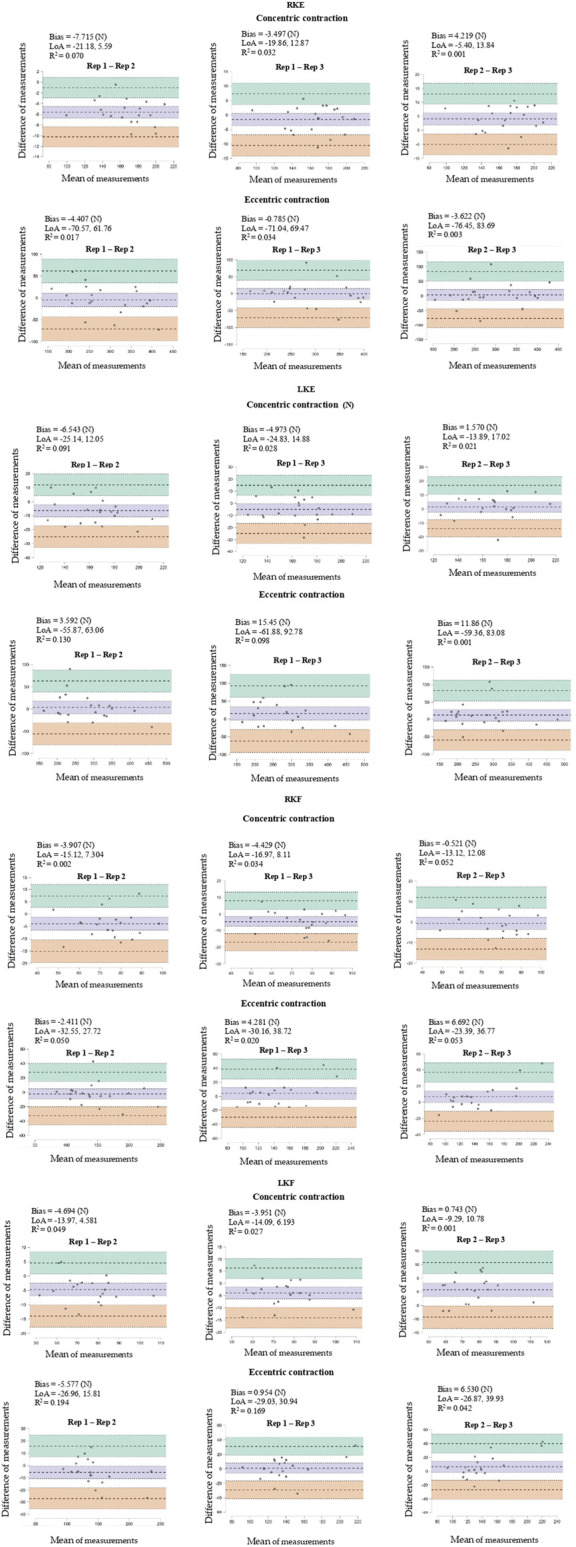
Bland-Altman test-retest plots for maximal muscle strength for right knee extension (RKE), left knee extension (LKE), right knee flexion (RKF), and left knee flexion (LRF).

The Bland-Altman plots reveal a low systematic bias (−7.715–15.45 N) for the assessment of peak muscle strength RKE, peak muscle strength right LKE, peak muscle strength RKF, peak muscle strength LKF in concentric and eccentric phase, in addition, a coefficient of determination *R* ^2^ = 0.194–0.001 as shown in Figure 2.

**FIGURE 3:**
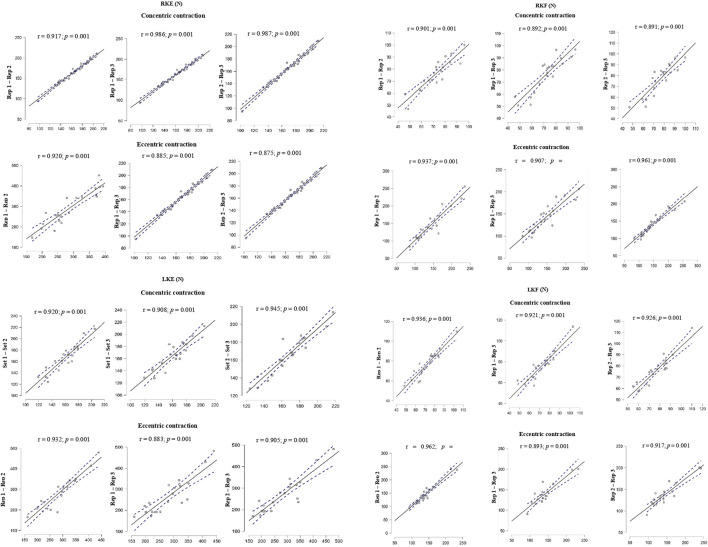
Level of association between maximum muscle strength in three repetitions for the assessment of peak muscle strength of knee movements: right knee extension (RKE), left knee extension (LKE), right knee flexion (RKF) and left knee flexion (LKF) in a concentric and eccentric displacement phase controlled by FEMD.

In the correlation analysis of muscle strength assessment, the magnitude r was almost perfect (0.908–0.987) for RKE, LKE and LKF very large for RKF (0.891–0.901) in the concentric phase. In the eccentric phase, the magnitude r was almost perfect for RKF (0.907–0.961), for RKE, LKE and LKF it was very large (0.875–0.962), all results were highly significant (*p* = 0.001). As shown in Figure 3.

## 4 Discussion

The purpose of this study was to analyze the relative and absolute reliability of intra-session comparisons of 3 repetitions in a peak muscle strength assessment protocol in a knee extension and flexion exercise in female soccer players. The results of our study confirm that the assessment of maximum muscle strength in knee flexion and extension for three repetitions, both concentrically and eccentrically, with FEMD is reliable.

In the study by Morenas-Aguilar et al. (2024), reliability measures similar to those of the present study were observed, reporting the reliability of 3 specific tests of peak isometric muscle strength (right and left foot elevation test and unilateral pullover) in fourteen handball players with FEMD, presenting an absolute reliability between CV = 7.85–11.57% and a relative reliability between ICC = 0.85–0.91. Similar results were obtained in a study Rodríguez-Perea et al. (2023), who evaluated the reliability of trunk rotators with FEMD, observing a high absolute (CV = 8.71–13.66%) and relative (ICC = 0.69–0.74) reliability in maximum muscle strength in the horizontal cable woodchop (HCW) at a speed of 40 m·s^-1^. When the speed of the evaluation was increased to 0.6 m·s^-1^, an absolute reliability (CV = 9.71–15.66%) and a relative reliability (ICC = 0.56–0.78) were reported, which is lower than that presented in our study. They also evaluated the trunk rotators with the low cable woodchop (LCW) exercise, presenting an absolute reliability (CV = 15.87–22.14%) and an absolute reliability ICC = (0.62–0.82) at a speed of 0.50 m·s^-1^, in addition, they reported an absolute reliability (CV = 14.32–18.96%) and an absolute reliability ICC = (0.75–0.84) at a speed of 0.70 m·s^-1^, which are much lower than those of our study. These results can be explained by the speed of the isokinetic tests implemented; previously, studies were conducted that analyzed the speed in isokinetic tests (Hadzic et al., 2012; Zanca et al., 2011). The results of these investigations indicate that when evaluating muscle strength in the different varieties of isokinetic tests, speeds lower than 0.6 m·s^−1^ are usually the most accurate at the time of their evaluation (Zanca et al., 2011).

In the study by Martinez-Garcia et al., 2020 they evaluated the strength of the shoulder joint with FEMD and reported reliability values similar to this study. It presents an acceptable absolute reliability (CV = 8.27%) and a relative reliability classified as very high (ICC = 0.85) in the measurements obtained from the concentric phase. In addition, they obtained for the eccentric phase an acceptable absolute reliability (CV = 7.38%) and relative reliability (ICC = 0.81) using a speed of 0.3 m·s^-1^. Likewise, an acceptable absolute reliability was reported (CV = 6.31%) and for the relative reliability, measures were obtained that are classified as extremely high (ICC = 0.93) in the concentric phase and for the eccentric phase, an absolute reliability (CV = 6.87%) and a very high relative reliability (ICC = 0.87) were recorded at a displacement speed of 0.6 m s-1 for the muscles that perform internal rotation of the shoulder. For shoulder muscles that generate external rotation of the shoulder, an acceptable absolute reliability measure was recorded (CV = 6.91%) and for the relative reliability measure it was classified as very high (ICC = 0.98) for the concentric movement phase, and the eccentric phase obtained an absolute reliability measure that is classified as acceptable (CV = 6.39%) and a relative reliability measure that is classified as very high (ICC = 0.90) at the displacement speed of 0.3 m·s^-1^. In relation to the concentric movement phase, the results obtained for the absolute reliability measurement were considered acceptable measurements (CV = 6.26%) and reported high measurements for relative reliability (ICC = 0.89), the eccentric phase presented an absolute reliability measurement classified as high (CV = 5.12%) and the relative reliability obtained a measurement that is classified as extremely high (ICC = 0.92) at a displacement speed of 0.6 m·s^-1^. The higher reliability of the concentric phase assessments in the evaluation of concentric muscle strength may be explained by the fact that the eccentric phase of sports movements is not practiced as frequently as the concentric phase (Nugent et al., 2015).

Although this study demonstrated the high intrasession reliability of the FEMD, future research should consider some limitations. Since we only analyzed female soccer players with a certain level of training, competition, and strength development, it is not possible to generalize our findings to other female athletes. Future studies should be initiated that can analyze intrasession reliability in different athletes and motor gestures for each particular sport, in addition, studies in people with reduced mobility and chronic pathologies.

## 5 Conclusion

The result of our study concludes that the evaluation of maximum muscular strength in knee flexion and extension in three repetitions in the concentric phase and eccentric phase with FEMD has a good relative and absolute reliability when evaluating professional female soccer players. This type of evaluation procedure helps to record the progress and development of female soccer players by providing reliable information in the recording of muscular strength. In addition, it demonstrates that intra-series measurements of FEMD are reliable when evaluating muscular strength.

## Data Availability

The raw data supporting the conclusions of this article will be made available by the authors, without undue reservation.
